# Phenotype-specific white matter microstructural alterations and their clinical correlates in Wilson disease

**DOI:** 10.1038/s41598-026-65470-z

**Published:** 2026-08-06

**Authors:** Ann Carolin Hausmann, Silja K. Querbach, Christian Rubbert, Alfons Schnitzler, Julian Caspers, Christian Johannes Hartmann

**Affiliations:** 1 Institute of Clinical Neuroscience and Medical Psychology, Medical Faculty and University Hospital Düsseldorf, Heinrich-Heine-University Düsseldorf, Moorenstr. 5, 40225 Düsseldorf, Germany; 2 Department of Diagnostic and Interventional Radiology, Medical Faculty and University Hospital Düsseldorf, Heinrich-Heine-University Düsseldorf, Moorenstr. 5, 40225 Düsseldorf, Germany; 3 Department of Neurology, Medical Faculty and University Hospital Düsseldorf, Heinrich-Heine-University Düsseldorf, Moorenstr. 5, 40225 Düsseldorf, Germany

**Keywords:** Wilson disease, Diffusion MRI, NODDI, Microstructure, Biomarkers, White matter, Biological techniques, Neurology, Neuroscience

## Abstract

Wilson disease (WD) is a genetic disorder of copper metabolism that leads to progressive brain damage, yet the microstructural mechanisms underlying white matter alterations remain insufficiently understood. Diffusion tensor imaging provides limited biological specificity and is sensitive to free water contamination. Therefore, we applied neurite orientation dispersion and density imaging to characterize white matter microstructure and its clinical relevance in 30 patients with WD, including neurological and hepatic phenotypes, and 30 matched healthy controls. We demonstrate widespread, phenotype-specific microstructural alterations, with reductions in neurite density and orientation dispersion accompanied by increased extracellular volume fraction and diffusivity metrics (mean, axial, and radial diffusivity) in neurological WD and isolated increased isotropic volume fraction without abnormalities in conventional diffusion metrics in hepatic WD across major white matter tracts. These patterns provide a coherent explanation for previously inconsistent fractional anisotropy findings. Reduced neurite density was associated with greater neurological impairment and lower cognitive performance, particularly in processing speed and visual attention. Together, these findings highlight neurite density as a potential marker of clinically relevant white matter disruption and reveal distinct microstructural signatures across WD phenotypes, consistent with differential underlying mechanisms and a potential progression from free water-related alterations to axonal degeneration. Advanced diffusion imaging thus enables a more specific characterization of white matter pathology and supports linking microstructural alterations to clinical outcomes in neurological disorders.

**Supplementary Information:**

The online version contains supplementary material available at 10.1038/s41598-026-65470-z.

## Introduction

Wilson disease (WD) is a rare autosomal-recessive disorder of impaired copper metabolism that leads to pathological copper accumulation in the liver and brain, and may result in hepatic dysfunction, neurological impairment, and cognitive deficits^1^. The most affected cognitive domains are memory, attention, processing speed, verbal fluency, and executive function^2–5^. Neurological manifestations in WD are associated with poorer prognosis and significantly reduced health-related quality of life compared with the hepatic form of the disease, largely attributable to cognitive decline^6,7^. Cerebral copper accumulation induces neurotoxic damage in the brain, primarily affecting deep gray matter nuclei and white matter (WM) tracts^7,8^. While a growing body of research has documented the clinical significance of basal ganglia impairments in WD^9–12^, only a limited number of studies have investigated the relationship between WM integrity and clinical symptoms^4,13,14^. Thus, the role of WM alterations in WD-related neurological and cognitive impairment remains poorly understood.

The evaluation of WM in WD is predominantly based on diffusion tensor imaging (DTI)^15^, which characterizes brain microstructure using diffusion-weighted magnetic resonance imaging (MRI) by quantifying the directional motion of water molecules^16^. These studies have reported widespread disruption in fiber integrity in WD, primarily reflected by reduced fractional anisotropy (FA) and increased mean diffusivity (MD), measuring the overall directionality and magnitude of water diffusion, respectively^13,14,17,18^. Despite the high sensitivity of DTI metrics to WM damage, their limited biological specificity leaves the underlying pathomechanisms unclear^19^. In addition, a recent study demonstrated increased free water content in the WM of WD patients and a marked attenuation of DTI abnormalities following free water correction^20^. Free water contamination, arising from partial volume effects between, e.g., cerebrospinal fluid and brain parenchyma within a voxel, may bias DTI measurements and contribute to inconsistencies in DTI findings across WD studies, particularly regarding the direction of FA changes^15^. Given its lack of specificity and susceptibility to partial volume effects, especially in regions with complex fiber architecture or inter-tissue interactions,^21,22^ conventional DTI may be insufficient to fully characterize WM pathologies in WD.

Recent advancements in diffusion MRI modeling have introduced neurite orientation dispersion and density imaging (NODDI)^23^ as a promising candidate for the development of next-generation imaging biomarkers of neuronal microstructure impairment^24,25^. In contrast to the single-tensor model used in DTI, NODDI decomposes the diffusion signal into three distinct tissue compartments^23^. Thereby, specific estimates of neurite density, neurite orientation dispersion, and free water proportion are derived, which are expected to provide biologically more meaningful information^23,24^. Application of NODDI has yielded encouraging results in clinical research, demonstrating high sensitivity and plausible interpretations of disease-related effects, as well as robust correlations with disease severity in patients with Alzheimer’s or Parkinson’s disease^24^. Preliminary NODDI studies in WD patients indicate significant microstructural impairment in the basal ganglia, thalamus,^9,10^ and WM^26^. However, the current evidence is scarce, particularly with respect to phenotype-specific changes, and the relationship between NODDI-derived WM metrics and clinical symptoms has not yet been investigated in WD.

With this study, we aimed to advance the currently limited knowledge by characterizing NODDI-derived microstructural WM alterations in WD patients with distinct clinical phenotypes and investigating their relationships with neurological and cognitive symptoms.

## Results

### Sample demographics and clinical characteristics

Five patients did not complete the MRI acquisition due to technical issues or discomfort, and one patient had an incidental finding. The final sample therefore included 30 WD patients, thereof 19 with a neurological phenotype (neuro-WD) and 11 with a hepatic phenotype (hep-WD), and 30 healthy controls. Detailed demographic and clinical characteristics are summarized in Table 1. There were no significant differences among the groups in terms of demographic variables, disease duration, or decoppering treatment. None of the participants showed signs of hepatic encephalopathy or had undergone liver transplantation.

**Table 1:** Data is either presented as counts, mean (± standard deviation), or median [interquartile range]. Significant group differences are highlighted in bold, with * = *p*-values < 0.05 and ** = *p*-values < 0.001.

	Neuro-WD	Hep-WD	Healthy controls	*p*
*Demographics*				
*n*	19	11	30	–
Male/female ratio	5/14	3/8	8/22	> 0.999
Age, years	43.5 (± 11.4)	34.3 (± 12.3)	40.1 (± 12.3)	0.143
Education, years	15.6 (± 2.5)	15.2 (± 2.7)	14.8 (± 1.9)	0.495
Disease duration, years	24.2 (± 11.0)	17.5 (± 9.4)	–	0.102
Treatment				
D-penicillamine	8	4	–	0.733
Trientine	10	5		
Chelator and zinc	1	2		
*Neurological score*				
UWDRS-N	6 [4–15]	2 [0–4]	0 [0–0]	< 0.001**
*Neuropsychological scores*				
MoCA	28 [25–29]	28 [26–28]	29 [28–29.25]	0.002*
SDMT, *z*	−0.55 (± 0.85)	0.10 (± 0.87)	0.55 (± 0.79)	< 0.001**
TMTA, *z*	0.39 [0.08–0.70]	0.38 [−0.38–1.15]	0.42 [0.03–0.84]	0.848
TMTB, *z*	0.59 [0.25–0.78]	0.24 [−0.35–0.99]	0.91 [0.50–1.30]	0.031*
WLMT-L, *z*	−0.28 (± 1.38)	−0.42 (± 1.17)	0.40 (± 0.78)	0.035*
WLMT-R, *z*	0.74 [0.21–1.26]	0.35 [−0.24–0.94]	1.03 [0.35–1.41]	0.154
SFT, *z*	0.12 (± 1.0)	−0.17 (± 1.30)	0.42 (± 0.84)	0.222

First, group differences in clinical scores were assessed to characterize the clinical symptoms in the present study sample. Patients with neuro-WD demonstrated significantly higher Unified Wilson’s Disease Rating Scale—neurological subscale (UWDRS-N) scores than both hep-WD patients (*p* = 0.004) and healthy controls (*p* < 0.001). Based on UWDRS-N subscores^27^, the most prevalent neurological symptoms were parkinsonism (68%), tremor (53%), and ataxia (42%), followed by dysarthria (32%), impaired handwriting (32%), dystonia (26%), rigidity (26%), and chorea (5%). Montreal Cognitive Assessment (MoCA) scores were significantly lower in both neuro-WD patients (*p* = 0.006) and hep-WD patients (*p* = 0.026) compared with healthy controls. Additionally, patients with neuro-WD performed worse than healthy controls on the Symbol Digit Modalities Test (SDMT; *p* < 0.001). Although a substantial difference in Trail Making Test Part B (TMTB) and Word List Memory Test list learning (WLMT-L) scores was found across the three groups, no specific pairwise differences were identified in post hoc analyses after correcting for multiple testing. No significant differences in Trail Making Test Part A (TMTA), Word List Memory Test delayed recall (WLMT-R), and semantic fluency test (SFT) performances were observed between groups. Except for the SDMT (*r*_*s*_ = −0.52, *p* = 0.023), we found no correlations between neuropsychological and UWDRS-N scores in WD patients.

### Differences in diffusion metrics: neuro-WD patients vs. healthy controls

**Fig. 1:**
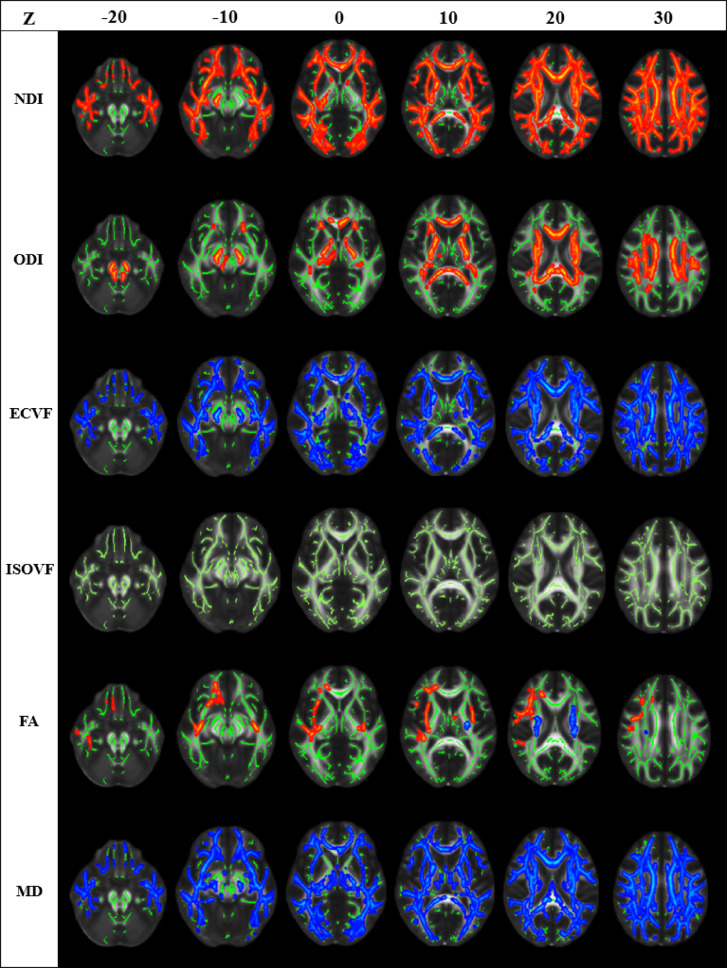
Differences in diffusion metrics between neuro-WD patients and healthy controls. WM regions with significant clusters of decreased (red-yellow) or increased (blue) diffusion metrics in neuro-WD patients compared with healthy controls are shown on the study-specific mean WM skeleton (green) on the FMRIB58 FA template (TFCE, FWE-corrected *p* ≤ 0.05). Results are presented on representative axial slices, ranging from Z = −20 to 30 (in MNI coordinates). The TBSS fill script was used to aid visualization. Images are displayed in accordance with radiological convention, meaning that the left side of the images corresponds to the right hemisphere of the brain and vice versa.

To characterize WM microstructural alterations in WD, we assessed group differences in diffusion MRI-derived NODDI and DTI metrics across the WM skeleton. *F*-Tests revealed significant differences in all diffusion metrics among at least one pair of groups (all family-wise error [FWE]-corrected *p* ≤ 0.05). Subsequent pairwise comparisons yielded significant alterations in neurite density index (NDI), orientation dispersion index (ODI), extracellular volume fraction (ECVF), and conventional diffusion metrics (FA, MD, axial diffusivity [AD], and radial diffusivity [RD]) in neuro-WD patients within extensive, symmetrical WM regions compared with healthy controls, whereas isotropic volume fraction (ISOVF) remained unchanged (all FWE-corrected *p* ≤ 0.05; see Fig. 1 and Fig. S1). Cluster-specific statistics are provided in Supplementary File 1 (see Tables S1–S7).

For both NDI and ODI, reductions involved widespread bilateral WM, affecting major commissural, projection, and association tracts, including the corpus callosum, corona radiata, and superior longitudinal fasciculus. Beyond this shared distribution, additional NDI reductions involved limbic WM, whereas ODI reductions additionally extended to brainstem projection pathways. Increases in ECVF, MD, AD, and RD showed a highly similar spatial distribution to the NDI reductions. FA decreases were found within the bilateral external capsule and right anterior corona radiata, retrolenticular part of internal capsule, superior longitudinal fasciculus, and sagittal stratum, whereas FA increases were restricted to the bilateral superior corona radiata and left posterior limb of internal capsule. While decreases in FA were primarily accompanied by reductions in NDI, rather than changes in ODI, increases in FA were predominantly observed in WM areas that demonstrated parallel reductions in NDI and ODI.

### Differences in diffusion metrics: hep-WD patients vs. healthy controls

**Fig. 2:**
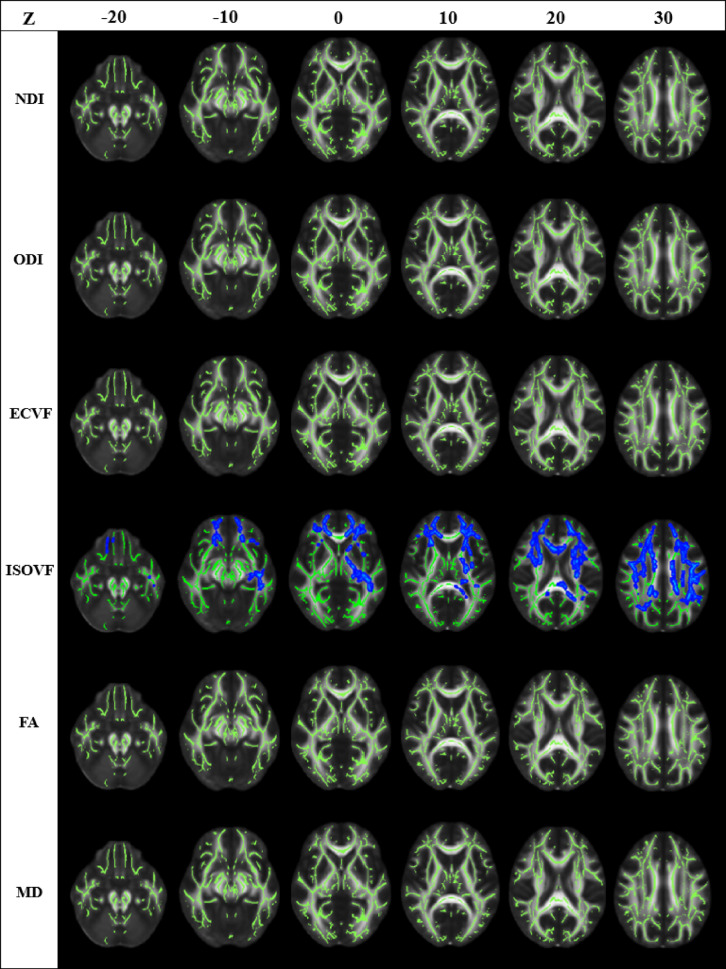
Differences in diffusion metrics between hep-WD patients and healthy controls. WM regions with significant clusters of increased ISOVF (blue) in hep-WD patients compared with healthy controls are shown on the study-specific mean WM skeleton (green) on the FMRIB58 FA template (TFCE, FWE-corrected *p* ≤ 0.05). Results are presented on representative axial slices, ranging from Z = −20 to 30 (in MNI coordinates). The TBSS fill script was used to aid visualization. The images are displayed in accordance with radiological convention, meaning that the left side of the images corresponds to the right hemisphere of the brain and vice versa.

Patients with hep-WD showed no differences in NDI, ODI, ECVF, FA, MD, AD, or RD compared with healthy controls. In contrast, ISOVF was significantly increased within the corpus callosum, bilateral anterior and superior corona radiata, bilateral superior longitudinal fasciculus, left external and internal capsule, and sagittal stratum (all FWE-corrected *p* ≤ 0.05; see Fig. 2 and Table S8 in Supplementary File 1).

### Differences in diffusion metrics: neuro-WD vs. hep-WD patients

**Fig. 3:**
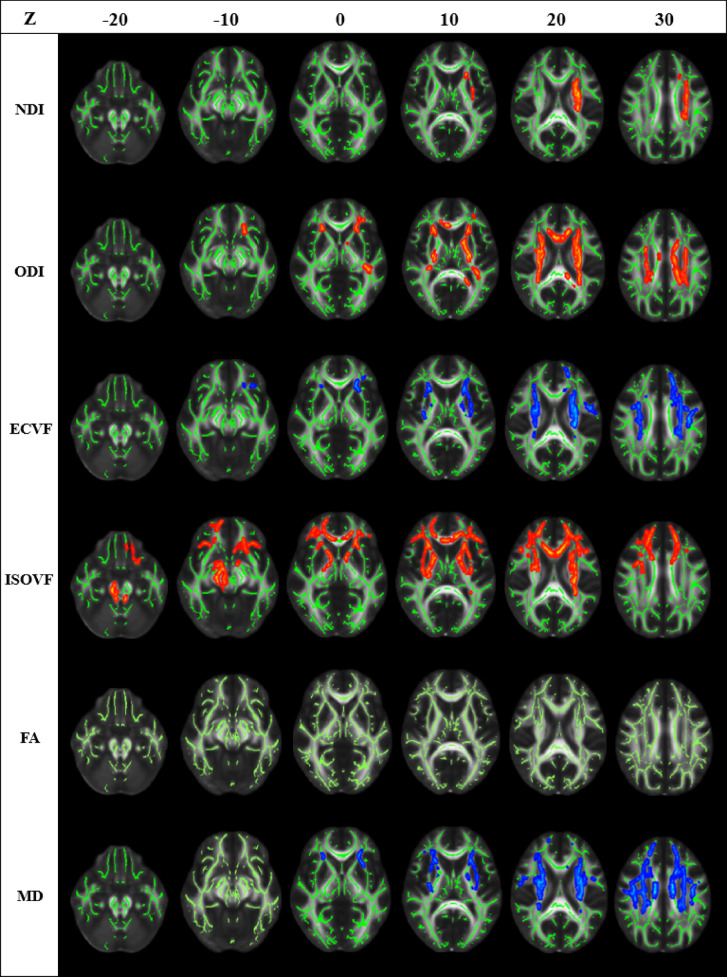
Differences in diffusion metrics between neuro-WD and hep-WD patients. WM regions with significant clusters of decreased (red-yellow) or increased (blue) diffusion metrics in neuro-WD patients compared with hep-WD patients are shown on the study-specific mean WM skeleton (green) on the FMRIB58 FA template (TFCE, FWE-corrected *p* ≤ 0.05). Results are presented on representative axial slices, ranging from Z = −20 to 30 (in MNI coordinates). The TBSS fill script was used to aid visualization. The images are displayed in accordance with radiological convention, meaning that the left side of the images corresponds to the right hemisphere of the brain and vice versa.

When comparing WD phenotypes, we found that patients with neuro-WD demonstrated significant reductions in NDI and ODI together with increased ECVF and MD, whereas those with hep-WD showed increased ISOVF (all FWE-corrected *p* ≤ 0.05; see Fig. 3). Similar differences were observed for AD, while no significant subgroup differences in RD or FA were detected (see Fig. 3 and S2). Cluster-specific statistics are provided in Supplementary File 1 (see Tables S9–S14). The affected WM regions overlapped with those identified in the comparisons with healthy controls, albeit the differences were less pronounced, particularly in NDI.

### Correlations with clinical scores

**Fig. 4:**
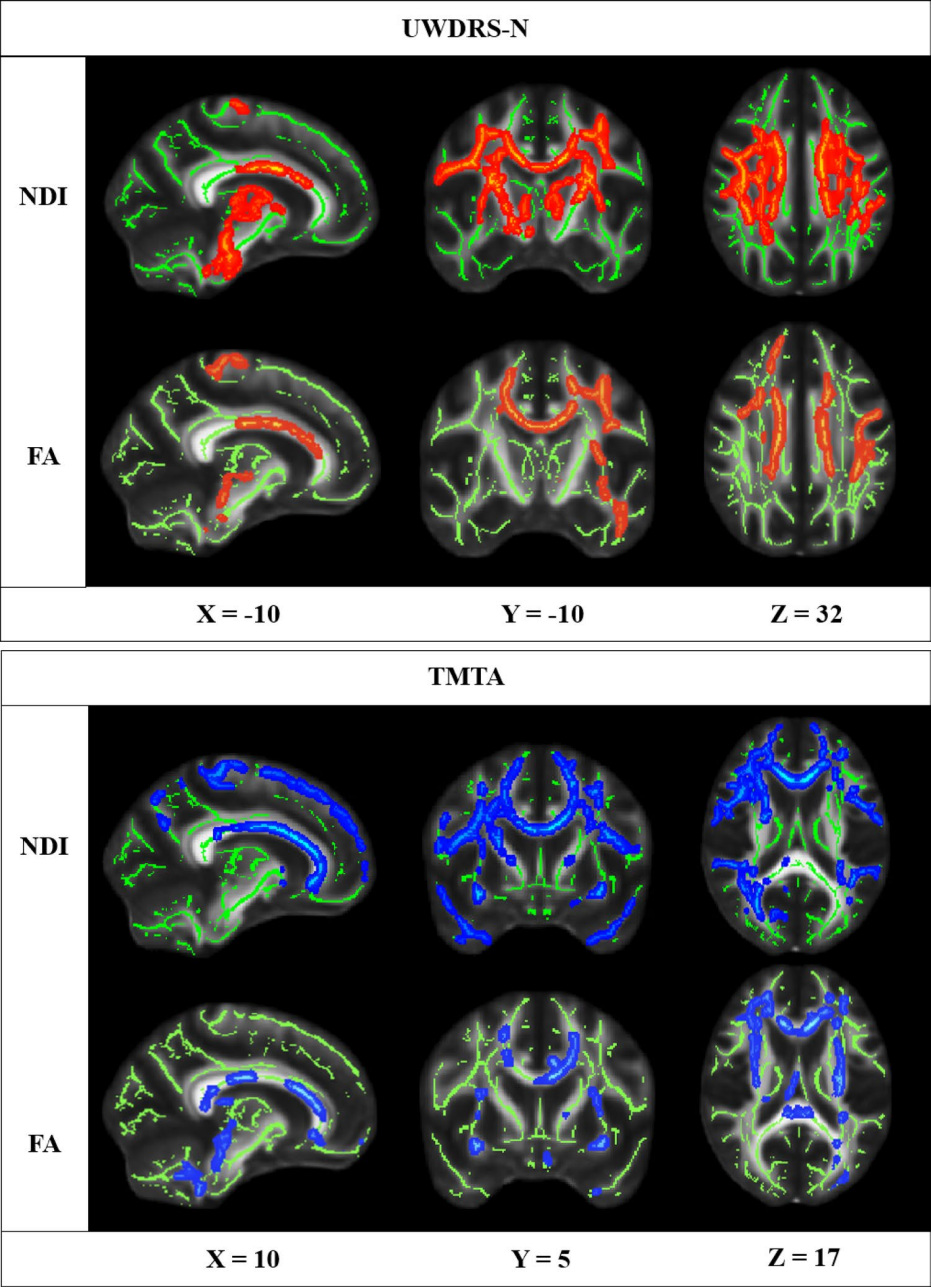
Significant associations between diffusion metrics and clinical scores. WM regions with significant clusters of decreased diffusion metrics with increasing UWDRS-N scores (red-yellow) or decreasing TMTA performance (blue; controlled for UWDRS-N scores) in WD patients are shown on the study-specific mean WM skeleton (green) on the FMRIB58 FA template (TFCE, FWE-corrected *p* ≤ 0.05). Results are presented on three representative slices (in MNI coordinates). The TBSS fill script was used to aid visualization. The images are displayed in accordance with radiological convention, meaning that the left side of the images corresponds to the right hemisphere of the brain and vice versa.

To assess clinical relevance, we next examined associations between the primary diffusion metrics and neurological and cognitive measures. Increased UWDRS-N scores were significantly correlated with NDI reductions within the corpus callosum, bilateral corona radiata, superior longitudinal fasciculus, external and internal capsule, posterior thalamic radiation, corticospinal tract, bilateral superior and middle cerebellar peduncles, and pontine crossing tract (all FWE-corrected *p* ≤ 0.05; see Fig. 4 and Table S15 in Supplementary File 1). Furthermore, poorer performance on the TMTA was significantly correlated with NDI reductions within the corpus callosum, bilateral corona radiata, superior longitudinal fasciculus, external and internal capsule, sagittal stratum, posterior thalamic radiation, fornix, and uncinate fasciculus (controlled for UWDRS-N scores; all FWE-corrected *p* ≤ 0.05; see Fig. 4 and Table S16 in Supplementary File 1). Reduced FA was significantly correlated with increased UWDRS-N scores, as well as poorer TMTA performance in most of the aforementioned WM regions, albeit to a lesser extent (all FWE-corrected *p* ≤ 0.05; see Fig. 4, Tables S19 and S20 in Supplementary File 1). We also identified WM tracts where NDI was decreased when performances on the SDMT or TMTB were diminished (see Fig. S3, Tables S17and S18 in Supplementary File 1), and where FA decreased with poorer performance on the TMTB (see Fig. S4and Table S21 in Supplementary File 1). However, once UWDRS-N scores were included as a covariate, these correlations were no longer significant. No associations between ODI or ISOVF and clinical scores were detected. Likewise, none of the examined diffusion metrics showed significant associations with the remaining neuropsychological scores.

## Discussion

This study systematically investigated WM alterations and their association with neurological and cognitive symptoms in patients with WD. We provide initial evidence for phenotype-specific differences in NODDI-derived WM microstructure, characterized by isolated increases in ISOVF in hep-WD patients and widespread reductions in NDI and ODI together with increased ECVF and diffusivity (MD, AD, and RD) in neuro-WD patients. Notably, NDI decreases across several major WM tracts were further identified as neuroimaging correlates of neurological impairment, processing speed, and visual attention. Therefore, this study offers novel insights into pathophysiological processes and underlines the clinical relevance of WM impairment in WD.

The observed NDI decreases in patients with neuro-WD resonate well with the preliminary results of Song et al., who reported widely reduced NDI in WD patients within WM regions that correspond to those identified in our study, including the corpus callosum, bilateral corona radiata, superior longitudinal fasciculus, superior fronto-occipital fasciculus, and thalamic radiation^26^. In contrast to their conference report, we additionally identified significant ODI reductions in these pathways and the brainstem. Microstructural abnormalities in the aforementioned fibers have also been identified in other neurodegenerative disorders with symptoms overlapping those of WD, such as tremor in Parkinson’s disease and chorea in Huntington’s disease^19^. Our results suggest global reductions in axonal density and a simplified fiber organization in neuro-WD patients compared with healthy controls. These observations are consistent with postmortem evidence of axonal loss, demyelination, cavitation, and tissue spongiosis in WD^7,28^, thereby supporting the biological plausibility of our findings. The concomitant increase in ECVF further supports the interpretation that reduced NDI is accompanied by expansion of the extra-neurite compartment, consistent with chronic tissue degeneration in neuro-WD. Likewise, the widespread increases in MD, AD, and RD are in line with previous DTI studies reporting increased diffusivity in WD^13,14^. In light of the concomitant reductions in NDI and ODI together with increased ECVF, these findings suggest reduced microstructural barriers to water diffusion accompanying progressive WM damage. Together, these complementary NODDI and DTI findings provide converging evidence for widespread chronic microstructural degeneration in neuro-WD.

Furthermore, we identified significantly increased ISOVF across diffuse WM regions in patients with hep-WD, which were previously only reported in the corpus callosum of WD patients^26^. NDI, ODI, ECVF, and all conventional DTI metrics (FA, MD, AD, and RD) remained unchanged compared with healthy controls, indicating preserved neurite density, orientation dispersion, extra-neurite volume, and diffusivity despite increased free water content. This pattern suggests that the observed ISOVF increase is unlikely to reflect tissue degeneration or atrophy, but rather represents an isolated increase in extracellular free water. A similar perspective is shared by Jing et al., who recently observed an increase in free water using bi-tensor free water imaging in hep-WD patients in WM regions overlapping with those identified in our study, including the corpus callosum, superior longitudinal fasciculus, anterior limb of internal capsule, and large parts of the corona radiata^20^. The DTI abnormalities in their hep-WD patients disappeared following free water correction^20^, suggesting a closer link to neuroinflammation rather than atrophy. In accordance with this hypothesis, astrocytes initially buffer elevated copper concentrations in the brain, resulting in a disruption in ionic homeostasis, cellular swelling, increased oxidative stress, and the activation of inflammatory responses^1,29^. One of the earliest consequences of cerebral copper overload is, thus, hydropic swelling of astrocytes and myelin sheaths, as well as astrogliosis^1,7^. In light of these considerations, the increased ISOVF observed in this study presumably reflects the presence of subclinical edema or neuroinflammation in the WM of hep-WD patients. The absence of ISOVF alterations in neuro-WD patients may be attributable to the recent observations in molecular studies indicating that chronic copper accumulation exceeds the buffering capacity of astrocytes, resulting in the exhaustion of their neuroprotective function and eventual death through cuproptosis^30^. Nevertheless, another contributing factor to our findings could be persistent hepatic dysfunction and subsequent portosystemic shunting of ammonia, causing neuropathological abnormalities that resemble those induced by copper^1,28^. This viewpoint is challenged by the absence of signs of hepatic encephalopathy in the examination of our patients and a previous NODDI study that did not identify ISOVF changes in patients with minimal hepatic encephalopathy compared with healthy controls^31^. However, it should be noted that the clinical criteria for hepatic encephalopathy are rather broad, meaning that more subtle signs may be missed.

The comparison of WD subgroups yielded a similar, but more subtle, pattern of findings than when each WD group was compared with healthy controls. Across all altered diffusion metrics, the overall anatomical pattern of affected WM tracts closely resembled that observed in the comparisons with healthy controls, although the extent of significant alterations was considerably reduced. Together, these findings support the concept of a gradual transition in WM involvement across the hepatic and neurological phenotypes of WD. Beyond these quantitative differences, the diffusion metrics revealed distinct microstructural signatures between the phenotypes. Whereas hep-WD was characterized exclusively by increased ISOVF, neuro-WD consistently demonstrated reduced NDI and ODI together with increased ECVF and diffusivity metrics. Although previous studies comparing hepatic and neurological WD phenotypes using conventional DTI have reported inconsistent findings, ranging from no significant differences in diffusion metrics^27^ to altered FA or increased diffusivity in neuro-WD^20,32^, the present multimodal analysis revealed phenotype-specific microstructural alterations across complementary diffusion metrics. This pattern suggests that free water accumulation may precede measurable expansion of the extra-neurite compartment and tissue degeneration. This hypothesis is consistent with the premise that copper exposure triggers a cascade of inflammatory pathways that ultimately leads to neurodegeneration^33^. Furthermore, the differences between neuro-WD and hep-WD patients in NDI were less pronounced than in ODI, which indicates that axonal loss may be initiated at an earlier stage of cerebral copper deposition than a reduction in axonal complexity. Taken together, our combined diffusion MRI findings suggest that NODDI could be a useful tool to assess the transition of WM alterations in WD from a potentially reversible edematous or neuroinflammatory state to axonal degeneration as the disease progresses from a hepatic to a neurological phenotype, even before becoming clinically evident. To clarify the role and temporal trajectory of these changes, longitudinal studies using NODDI should be conducted in patients with different disease states, including treatment-naïve patients, in addition to the assessment of laboratory copper indices.

Consistent with previously reported inconsistencies in FA findings across WD studies^15^, we observed FA alterations in both directions in neuro-WD patients. A decrease in FA was primarily observed in WM tracts with decreased NDI but unchanged ODI, while an increase in FA was observed in regions with parallel decreases in NDI and ODI. On the one hand, this finding indicates that the observed reductions in FA were primarily attributable to decreased axonal density and reflect a genuine loss of WM integrity within the bilateral external capsule, as well as the right anterior corona radiata, retrolenticular part of internal capsule, superior longitudinal fasciculus, and sagittal stratum. This aligns with previous studies^13,18,32^. On the other hand, the concomitant presence of reduced axonal density and fiber dispersion may paradoxically increase FA, which can be misinterpreted if ODI is not considered. A similar observation was reported in the WM of Alzheimer’s disease patients by Douaud et al. (2011), who proposed that the misleading FA increase could be attributable to the selective degeneration of individual fiber populations within crossing fibers. This may result in a less diverse pattern in orientation dispersion and, consequently, in an apparently increased alignment of the preserved axons^24,34^. Our study found that FA was increased significantly within the superior corona radiata and the posterior limb of the internal capsule, which are known for their complex fiber architecture^35^. Thus, we recommend that future WD studies examine potential selective damage to crossing fibers within these key regions more thoroughly by employing targeted techniques, such as fixel-based analysis or probabilistic tractography^36,37^. In summary, our findings provide plausible explanations for the bidirectional FA alterations in WD by demonstrating how NODDI can disentangle the respective contributions of axonal loss and changes in fiber organization.

Significant correlations were identified between increased UWDRS-N scores and decreased NDI in motor-related WM tracts. Our findings align with previously reported correlations between neurological symptoms and microstructural alterations within the corticospinal tract^14^, left internal capsule, thalamic radiation, corpus callosum^27^, and superior cerebellar peduncles in WD^20^. The corpus callosum, the principal WM fiber bundle in the brain, was found to be consistently affected in all our analyses. Callosal microstructural integrity is associated with interhemispheric communication, motor control, and the coordination of bimanual motor tasks^38,39^. In accordance with our findings, a previous study has demonstrated that WD patients with corpus callosum abnormalities exhibited more severe neurological dysfunction compared to patients without such lesions^40^. In addition, we identified a correlation between neurological symptoms and microstructural alterations within the corona radiata and internal capsule, including thalamic radiation and corticospinal tract fibers, which form large, interconnected pathways that link the cerebral cortex, thalamus, and brainstem. Fibers within the internal capsule also traverse the basal ganglia, and fiber volumes between the putamen and thalamus have been demonstrated to be lower in WD patients who present with hypokinesia^41^. Given that the brain regions interconnected by the compromised fibers identified in our study have previously been implicated in the neurological severity of WD^27,42,43^, we hypothesize that a reduction in axonal density may further exacerbate motor dysfunction in patients with WD by hampering connectivity between these structures. This would complement the findings of previous studies, which have indicated that extrapyramidal symptoms in WD can be attributable to structural and functional abnormalities within the cerebello-thalamo-cortical network^41,44,45^. Overall, our findings suggest that axonal degeneration in several large WM pathways, such as the corpus callosum, internal capsule, and corona radiata, may contribute to the manifestation of more severe neurological symptoms in patients with WD.

Both WD groups performed worse on the MoCA than healthy controls, and neuro-WD patients also had significantly lower SDMT scores. We additionally found group differences in TMTB and WLMT-L performances that could not be further resolved in post hoc analyses, presumably due to the limited statistical power resulting from small sample sizes. Our findings align with those of earlier studies that reported a mild decline in global cognitive function, attention, processing speed, executive function, and verbal learning in patients with WD^2–5^. Overall, the microstructural alterations identified in this study also compromised WM fibers integral to high-level cognitive functions. In addition to its role in motor function, the corpus callosum is considered essential to executive and global cognitive function, with the genu contributing significantly to working memory^46^. Likewise, the microstructural integrity of the corona radiata has been linked to multiple dimensions of attention in healthy individuals^47^, while the superior longitudinal fasciculus plays a critical role in integrating information underlying complex cognitive functions, including language processing, visuospatial attention, verbal working memory, and global executive function^46,48^. In line with these considerations, we identified significant correlations between reduced NDI in these regions and poorer performances on the TMTA, TMTB, and SDMT. However, only the correlation with TMTA performance persisted after controlling for UWDRS-N scores, suggesting that subtle dysarthria and mildly impaired handwriting ability may have confounded TMTB and SDMT performance through a reduction in motor speed. Therefore, our findings indicate that regional reductions in axonal density affect processing speed, visual attention, and/or visuomotor coordination in patients with WD. This aligns with the observation that, while multifocal cerebral pathologies tend to negatively affect cognitive performance in WD, they do not necessarily result in a significant deterioration of cognitive deficits beyond the impact of basal ganglia impairments^5^. Moreover, given that no correlations were found between clinical scores and ODI or ISOVF, the observed changes in fiber dispersion and free water probably did not impact neurological or cognitive function in our patients.

The present findings may have several implications. While both DTI and NODDI demonstrated WM impairment in neuro-WD patients, the extent of NODDI alterations was found to be more pronounced and plausibly explained the specific microstructural underpinnings of bidirectional FA scalars. Importantly, hep-WD patients exhibited an isolated increase in ISOVF despite preserved conventional DTI metrics, indicating that early WM alterations may remain undetected by tensor-derived diffusion measures. In addition, the identified increase in free water fraction in hep-WD patients can bias DTI-derived conclusions due to the model’s susceptibility to partial volume contamination. Therefore, we recommend the implementation of advanced diffusion MRI modeling with NODDI to complement DTI in future studies to more accurately assess WM microstructure in WD. In the past, DTI metrics have been proposed as useful markers in earlier stages of WD^15^, as they can trace brain abnormalities prior to the manifestation of morphological changes in structural MRI and clinically overt neurological symptoms^32^ and may improve following decoppering therapy^49^. Similarly, our findings extend previous work demonstrating the clinical relevance of structural MRI-based biomarkers in WD. In particular, the semiquantitative MRI severity scale proposed by Dusek et al., which distinguishes acute toxicity-related abnormalities from chronic damage, has been shown to correlate with serum neurofilament light chain (sNfL), a biomarker of neuroaxonal injury, as well as neurological severity, supporting the value of brain MRI for disease assessment and treatment monitoring in WD^50–52^. Building upon these established MRI biomarkers, our findings suggest that NODDI could offer additional value through its sensitivity to different progression stages of WM involvement across WD phenotypes, as indicated by its capacity to discern subclinical signs of cerebral copper toxicity in hep-WD as well as long-term axonal disruption in neuro-WD. NDI was identified as a potentially valuable candidate for the development of novel imaging markers, as we demonstrated that decreased axonal density may contribute to WD-related neurological impairment, as well as to lower processing speed and visual attention. Future longitudinal studies may offer further insights into the benefits of using NODDI-derived biomarkers in MRI evaluations to potentially assist in disease monitoring to adjust treatment regimens before clinically meaningful axonal degeneration manifests.

This study has several limitations. The limited sample size, particularly within phenotype subgroups, constrained statistical power; however, given the very low prevalence of WD, recruitment of eligible patients is inherently challenging, and the sample size of the present study is substantial compared with that of other neuroimaging studies in WD. Nevertheless, findings require replication in larger, independent cohorts. Neuro-WD patients in our study exhibited relatively low UWDRS-N scores, as severe neurological impairments precluded completion of the study assessments. Microstructural alterations in more severe cases may therefore have been underestimated. Given the cross-sectional design, the observed differences between phenotypes cannot be interpreted as evidence of longitudinal disease progression. However, the distinct microstructural patterns may reflect differential underlying mechanisms or stages of tissue involvement. From a methodological perspective, we further decided to employ the standard Watson-NODDI model, which has demonstrated reliable performance in clinical research^24^ and in initial WD studies^9,10^. Subsequent studies could extend these analyses by implementing the Bingham-NODDI extension, which additionally quantifies the anisotropic orientation dispersion of neurites^53^. Beyond alternative NODDI models, more recent fiber-specific diffusion approaches, including constrained spherical deconvolution and multi-fixel models, may further extend the present findings by disentangling fiber-specific alterations within regions of complex crossing architecture^36,54^, thereby complementing the compartment-specific information provided by NODDI. Furthermore, the TBSS framework restricts the analysis to the WM skeleton, which may reduce sensitivity to peripheral or tract-specific alterations outside the skeletonized representation^55^. Consequently, subtle microstructural abnormalities outside the core of major WM tracts may not have been captured. Future studies employing tract- or fiber-specific analyses may further refine the spatial characterization of WM pathology in WD. Moreover, because TBSS projects locally maximal FA values onto the skeleton, noise-related fluctuations may influence the projected diffusion metrics. Dedicated denoising was not part of the HCP-style preprocessing we used here^56^, which predates the introduction of MP-PCA denoising^57^, but not TBSS^55^, and the integration of denoising with HCP-based workflows remains an area of methodological discussion. Therefore, future work should evaluate denoising in sensitivity analyses beginning with the original reconstructed diffusion-weighted images. It should also be noted that diffusion MRI-derived metrics provide indirect, model-based estimates of tissue microstructure and should not be interpreted as direct measures of underlying histological properties. However, their spatial distribution and clinical associations support their biological relevance in this context. Finally, although there is increasing evidence for the potential of advanced diffusion MRI modeling, translating these novel techniques into clinical practice remains challenging and will require further validation.

In conclusion, this study has revealed phenotype-specific microstructural WM alterations in patients with WD, characterized by excess free water in hep-WD and global reductions in axonal density and fiber organization in neuro-WD. Our results suggest that NODDI could be a viable method for forecasting the conversion from edematous or neuroinflammatory states in hep-WD patients to clinically meaningful WM neurodegeneration in neuro-WD patients. Decreased neurite density primarily contributed to the severity of neurological impairment but also negatively affected processing speed and visual attention. Overall, NODDI-derived diffusion metrics may prove useful for monitoring disease progression and could serve as potential imaging biomarkers for the early detection of the conversion to neurological manifestations in WD.

## Methods

### Study participants

This study was part of a larger observational imaging study on neurodegenerative diseases (DRKS00034050). All procedures were performed in compliance with relevant laws, institutional guidelines, and the principles put forth in the Declaration of Helsinki in its latest revision and have been approved by the local ethics committee of the Medical Faculty at the Heinrich-Heine-University Düsseldorf, Germany (2019-470_4; 7th of February 2022). Written informed consent was obtained from all subjects prior to study participation. The privacy rights of all participants have been observed.

In this prospective, cross-sectional study, we recruited 36 WD patients from the WD outpatient clinic of the Department of Neurology of the University Hospital Düsseldorf, Germany. In addition, 30 age- and sex-matched healthy controls were enrolled. We included patients aged 18 years or older with a Leipzig score of ≥ 4, suggesting an established diagnosis of WD according to international consensus criteria^58^. Patients who initially presented with or developed significant neurological impairment before study enrollment were classified as neuro-WD. Those without significant neurological impairment at any stage of the disease were classified as hep-WD. The exclusion criteria encompassed contraindications to MRI, as well as the presence or history of any neurological disease other than WD or other severe medical conditions that would impede the completion of study assessments. Participants were also examined in a previous study that focused on investigating neuroimaging biomarkers related to gray matter atrophy^59^. In contrast, the current study evaluated microstructural alterations in global WM.

### Clinical assessments

Neurological symptoms were thoroughly assessed using the UWDRS-N by a senior neurologist in the field of movement disorders (CJH, 15 years of experience with WD). Additionally, standardized neuropsychological testing of multiple cognitive domains was conducted. The MoCA was administered to evaluate global cognitive function. Sustained attention and processing speed were tested using the oral version of the SDMT and the written form of the TMTA. Executive function was tested using the TMTB. Verbal memory was assessed using the WLMT-L and WLMT-R of the Consortium to Establish a Registry for Alzheimer’s Disease (CERAD) test battery and the SFT (animals). Cognitive scores were standardized via *z*-transformations using normative data.

### Imaging acquisition

High-resolution structural and multi-shell diffusion MRI data were acquired on a 3 T MRI scanner (MAGNETOM Prisma, Siemens Healthineers, Erlangen, Germany) using a 64-channel head coil. The protocol included the following sequences and parameters, which were adapted from the Lifespan Human Connectome Project (HCP) in Aging^60^: 3D T1-weighted magnetization-prepared rapid gradient-echo (MPRAGE) sequence (repetition time [TR] = 2,500 ms, echo time [TE] = 2.22 ms, inversion time [TI] = 1,000 ms, flip angle = 8°, field of view [FoV] = 240 × 256 mm, slice thickness = 0.8 mm) and diffusion-weighted spin-echo echo-planar imaging (EPI) (TR = 3,230 ms, TE = 89.2 ms, FoV = 210 × 210 mm, voxel size = 1.5 × 1.5 × 1.5 mm^3^, multi-band factor 3; 14 *b*_*0*_ images, 93 diffusion-encoding directions at *b* = 1500 s/mm^2^, and 92 diffusion-encoding directions at *b* = 3000 s/mm^2^). Foam padding was used to minimize head motion. Sequences were visually inspected for image quality during MRI acquisition. When pronounced motion artifacts resulting in visible image degradation were identified, the affected sequences were repeated within the same scanning session. No predefined quantitative motion threshold was applied.

### Processing of MRI data

The imaging data was preprocessed using the HCP minimal preprocessing pipelines (version 4.2.0)^56^ employing FreeSurfer (version 5.3) and the Oxford Centre for Functional MRI of the Brain (FMRIB) Software Library (FSL; version 6.0.2)^61^. Structural images were corrected for gradient nonlinearity-induced, readout, and bias field distortions, and were linearly and nonlinearly aligned to the Montreal Neurological Institute (MNI) 152 standard space and the anterior and posterior commissure plane^56^. Undistorted images were skull-stripped to extract brain tissue, followed by segmentation, surface reconstruction, and generation of a structural brain mask^56^. For the diffusion data, b_0_ intensity was normalized across timeseries and the phase-encoding direction-reversed b_0_ pairs were used to correct for EPI geometric distortion via FSL’s topup algorithm^62^. Eddy current and head motion correction was performed using the eddy tool^63^. Adhering to the HCP minimal preprocessing pipeline^56^, no dedicated diffusion MRI denoising step, such as Marchenko-Pastur principal component analysis (MP-PCA) denoising^57^, was applied. After gradient nonlinearity correction, the mean b_0_ images were registered to their corresponding T1-weighted images, and diffusion data were resampled and masked with the structural brain masks.

The tensor model was fitted using the dtifit command of FSL’s diffusion toolbox to generate maps of FA, MD, AD, and RD. FA reflects the degree of diffusional directionality, whereas MD quantifies the overall magnitude of water diffusion. AD and RD describe diffusion parallel and perpendicular to the principal diffusion direction, respectively.

The GPU-accelerated Compute Unified Device Architecture (CUDA) Diffusion Modelling Toolbox (cuDIMOT)^64^ within FSL was used to derive the following metrics of the Watson-NODDI model: (1) NDI, indicating the packing density of axons, (2) ODI, indicating the spatial organization of axons through variability in fiber orientation, and (3) ISOVF, indicating free water content^23^. An additional ECVF map, reflecting the extra-neurite compartment, was calculated from the fitted NODDI parameters according to the Watson-NODDI compartment model^23^: $ECVF=\left({1}-ISOVF\right)\times \left({1}-NDI\right)$, where ISOVF denotes the isotropic compartment fraction and NDI the intra-neurite compartment fraction.

### Tract-based spatial statistics

DTI and NODDI maps were analyzed using Tract-Based Spatial Statistics (TBSS)^55^ implemented in FSL using the standard TBSS pipeline and default processing steps. First, FA maps were registered to the FMRIB58 FA template in the MNI152 standard space using nonlinear transformations. Aligned FA data were averaged across subjects and thinned to generate a study-specific mean WM skeleton, which represents the center of all tracts that are common among our subjects^55^. The skeleton was thresholded at a mean FA of 0.2 to suppress non-WM voxels and those with poor tract correspondence. Each subject’s normalized FA map was projected onto the mean skeleton using the standard projection algorithm implemented in TBSS. For each voxel of the mean skeleton, the corresponding FA value was obtained from the local maximum identified in the aligned FA image along a direction perpendicular to the local WM tract^55^. To avoid assigning values from neighboring tracts, the search was constricted to the closest skeleton segment and incorporated the default TBSS soft distance weighting, which progressively downweights more distant voxels^55^. The FA-derived nonlinear warps and projection vectors were then applied to skeletonize the remaining DTI-derived maps (MD, AD, and RD) and all NODDI-derived maps (NDI, ODI, ISOVF, and ECVF) in the same manner for subsequent voxel-wise statistical analyses.

We created a one-way analysis of variance (ANOVA) design matrix using the general linear model to compare the diffusion maps between the neuro-WD (*n* = 19), hep-WD (*n* = 11), and healthy control groups (*n* = 30). An initial *F*-test, followed by pairwise *t*-contrasts, was defined. To explore the voxel-wise correlations between the primary diffusion metrics (NDI, ODI, ISOVF, and FA) and clinical symptoms in the WD patient cohort (*n* = 30), neurological and cognitive scores were added as independent variables. Where significant associations with cognitive scores were identified, subsequent analyses were performed, including UWDRS-N as a covariate. All defined models were controlled for the covariate effects of age and sex. Voxel-wise non-parametric permutation analyses with 5,000 permutations and Threshold-Free Cluster Enhancement (TFCE) were performed using FSL randomise. Results were considered significant at a family-wise error (FWE) corrected *p* ≤ 0.05. Significant clusters were extracted via the cluster tool in FSL and anatomically localized using the Johns Hopkins University ICBM-DTI-81 WM atlas.

### Statistical analysis

Shapiro–Wilk tests were used to evaluate normality. Group differences in demographic data (*n* = 60) were tested using Fisher-Freeman-Halton exact tests for frequencies and one-way ANOVA for continuous variables. Clinical scores were compared between neuro-WD patients (*n* = 19), hep-WD patients (*n* = 11), and healthy controls (*n* = 30) using ANOVAs or, for non-normally distributed or ordinal variables, Kruskal–Wallis tests, followed by Bonferroni or Dunn’s tests for post hoc pairwise comparisons. Spearman correlation coefficients were used to evaluate the relationship between neurological and neuropsychological scores in the patient cohort (*n* = 30). All statistical analyses were performed using SPSS Statistics (v29; IBM Corp, New York) with a level of significance set at *p* ≤ 0.05 (two-tailed). Bonferroni correction was employed to adjust for multiple comparisons.

## Supplementary Information

Below is the link to the electronic supplementary material.

Supplementary Material 1.

## Data Availability

The datasets generated and/or analyzed during the current study are not publicly available due to privacy and data protection regulations, but anonymized data may be requested from the corresponding author upon reasonable request.
