# Supplementary material for: Phenotype-specific white matter microstructural alterations and their clinical correlates in Wilson disease
Source: Sci Rep. 2026 Aug 6;16:24344. doi: 10.1038/s41598-026-65470-z (PMC13447857; doi:10.1038/s41598-026-65470-z)
Supplement: Supplementary file 1 [file 41598_2026_65470_MOESM1_ESM.docx]

Supplementary File 1

**1. Cluster-specific statistics: Group differences in diffusion metrics**

All supplementary tables report the cluster-specific statistics derived from TBSS analyses, including the peak MNI coordinates (corresponding to the voxel with the highest test statistic), total voxels, and minimum FWE-corrected *p*-value of each significant cluster. The peak coordinates are provided for anatomical reference only. WM tract localization was determined independently by assessing the overlap of the entire significant cluster with the JHU WM atlas. Consequently, a single significant cluster may encompass multiple WM tracts across one or both hemispheres. For each overlapping WM region, the corresponding voxel count and mean FWE-corrected *p*-values are reported. To mitigate the occurrence of false positives, only robust clusters and regions with an overlap of more than 100 voxels are displayed.

Abbreviations used in this file:

WM = white matter; NDI = neurite density index; ODI = orientation dispersion index; ECVF = extracellular volume fraction; ISOVF = isotropic volume fraction; FA = fractional anisotropy; MD = mean diffusivity; AD = axial diffusivity; RD = radial diffusivity; neuro-WD = Wilson disease patients with a neurological phenotype; hep-WD = Wilson disease patients with a hepatic phenotype; MNI = Montreal Neurological Institute; JHU = Johns Hopkins University; NODDI = Neurite Orientation Dispersion and Density Imaging; DTI = Diffusion Tensor Imaging; B = body; G = genu; S = splenium; R = right; L = left; M = middle; FWE = family-wise error; UWDRS-N = Unified Wilson’s Disease Rating Scale neurological subscale; TMTA = Trail Making Test Part A; TMTB = Trail Making Test Part B; SDMT = Symbol Digit Modalities Test; TBSS = Tract-Based Spatial Statistics; TFCE = Threshold-Free Cluster Enhancement.

1.1 **Neuro-WD patients vs. healthy controls**

**Table S1 WM regions with decreased NDI in neuro-WD patients compared with healthy controls**

| Peak MNI coordinates | | | WM regions | Side | Voxels | Total voxels | Mean *p* |
| --- | --- | --- | --- | --- | --- | --- | --- |
| X | **Y** | **Z** |  |  |  |  |  |
| 35 | 5 | -2 |  |  |  | 77,129 | *p_min_* < .001 |
|  | | | Corpus callosum | B | 2,758 | 5,391 | .008 |
|  |  |  |  | G | 1,465 |  |  |
|  |  |  |  | S | 1,168 |  |  |
|  | | | Superior corona radiata | R | 1,455 | 2,829 | .002 |
|  |  |  |  | L | 1,374 |  |  |
|  | | | Superior longitudinal fasciculus | R | 1,469 | 2,762 | .005 |
|  |  |  |  | L | 1,293 |  |  |
|  | | | Anterior corona radiata | R | 1,298 | 2,613 | .006 |
|  |  |  |  | L | 1,315 |  |  |
|  | | | External capsule | R | 1,014 | 2,076 | .006 |
|  |  |  |  | L | 1,062 |  |  |
|  | | | Posterior corona radiata | R | 587 | 1,129 | .008 |
|  |  |  |  | L | 542 |  |  |
|  | | | Posterior thalamic radiation | R | 604 | 959 | .020 |
|  |  |  |  | L | 355 |  |  |
|  | | | Anterior limb of internal capsule | R | 448 | 871 | .006 |
|  |  |  |  | L | 423 |  |  |
|  | | | Retrolenticular part of internal capsule | R | 429 | 722 | .012 |
|  |  |  |  | L | 293 |  |  |
|  | | | Posterior limb of internal capsule | R | 446 | 710 | .010 |
|  |  |  |  | L | 264 |  |  |
|  | | | Cingulum | R | 260 | 645 | .013 |
|  |  |  |  | L | 385 |  |  |
|  | | | Sagittal stratum | R | 319 | 593 | .014 |
|  |  |  |  | L | 274 |  |  |
|  | | | Superior fronto-occipital fasciculus | R | 106 | 203 | .002 |
|  |  |  |  | L | 97 |  |  |
|  | | | Fornix | R | 163 | 196 | .012 |
|  |  |  |  | L | 33 |  |  |
|  | | | Cerebral peduncle | R | 175 | 175 | .011 |
|  | | | Uncinate fasciculus | R | 75 | 132 | .007 |
|  |  |  |  | L | 59 |  |  |

*Note*. Cluster-specific TBSS results for the group comparison neuro-WD vs. healthy controls in NODDI-derived NDI. For more detailed explanation, see general description above.

**Table S2 WM regions with decreased ODI in neuro-WD patients compared with healthy controls**

| Peak MNI coordinates | | | WM regions | Side | Voxels | Total voxels | Mean *p* |
| --- | --- | --- | --- | --- | --- | --- | --- |
| X | **Y** | **Z** |  |  |  |  |  |
| -12 | -18 | -16 |  |  |  | 20,736 | *p_min_* < .001 |
|  | | | Corpus callosum | B | 2,552 | 5,852 | .003 |
|  |  |  |  | G | 1,275 |  |  |
|  |  |  |  | S | 2,025 |  |  |
|  | | | Superior corona radiata | R | 1,062 | 1,991 | .002 |
|  |  |  |  | L | 929 |  |  |
|  | | | Posterior limb of internal capsule | R | 640 | 1,362 | .002 |
|  |  |  |  | L | 722 |  |  |
|  | | | Superior longitudinal fasciculus | R | 634 | 932 | .006 |
|  |  |  |  | L | 298 |  |  |
|  | | | Anterior corona radiata | R | 481 | 922 | .003 |
|  |  |  |  | L | 441 |  |  |
|  | | | Anterior limb of internal capsule | R | 411 | 834 | .003 |
|  |  |  |  | L | 423 |  |  |
|  | | | Posterior corona radiata | R | 424 | 797 | .004 |
|  |  |  |  | L | 373 |  |  |
|  | | | Cerebral peduncle | R | 404 | 770 | .004 |
|  |  |  |  | L | 366 |  |  |
|  | | | Cerebellar peduncle | M | 510 | 510 | .020 |
|  | | | Retrolenticular part of internal capsule | R | 247 | 477 | .004 |
|  |  |  |  | L | 230 |  |  |
|  | | | Corticospinal tract | R | 159 | 394 | .005 |
|  |  |  |  | L | 235 |  |  |
|  | | | Superior cerebellar peduncle | R | 166 | 322 | .010 |
|  |  |  |  | L | 156 |  |  |
|  | | | Posterior thalamic radiation | R | 231 | 316 | .007 |
|  |  |  |  | L | 85 |  |  |
|  | | | External capsule | R | 76 | 225 | .005 |
|  |  |  |  | L | 149 |  |  |
|  | | | Medial lemniscus | R | 111 | 199 | .013 |
|  |  |  |  | L | 88 |  |  |
|  | | | Superior fronto-occipital fasciculus | R | 89 | 168 | .002 |
|  |  |  |  | L | 79 |  |  |

*Note*. Cluster-specific TBSS results for the group comparison neuro-WD vs. healthy controls in NODDI-derived ODI. For more detailed explanation, see general description above.

**Table S3 WM regions with increased ECVF in neuro-WD patients compared with healthy controls**

| Peak MNI coordinates | | | WM regions | Side | Voxels | Total voxels | Mean *p* |
| --- | --- | --- | --- | --- | --- | --- | --- |
| X | **Y** | **Z** |  |  |  |  |  |
| 35 | 4 | -1 |  |  |  | 71,798 | *p_min_* = .001 |
|  | | | Corpus callosum | B | 2,473 | 4,979 | .010 |
|  |  |  |  | G | 1,407 |  |  |
|  |  |  |  | S | 1,099 |  |  |
|  | | | Superior corona radiata | R | 1,378 | 2,714 | .003 |
|  |  |  |  | L | 1,336 |  |  |
|  | | | Superior longitudinal fasciculus | R | 1,311 | 2,458 | .008 |
|  |  |  |  | L | 1,147 |  |  |
|  | | | Anterior corona radiata | R | 1,177 | 2,351 | .007 |
|  |  |  |  | L | 1,174 |  |  |
|  | | | External capsule | R | 960 | 1,977 | .007 |
|  |  |  |  | L | 1,017 |  |  |
|  | | | Posterior corona radiata | R | 491 | 958 | .008 |
|  |  |  |  | L | 467 |  |  |
|  | | | Anterior limb of internal capsule | R | 444 | 859 | .006 |
|  |  |  |  | L | 415 |  |  |
|  | | | Posterior limb of internal capsule | R | 433 | 841 | .015 |
|  |  |  |  | L | 408 |  |  |
|  | | | Posterior thalamic radiation | R | 468 | 654 | .019 |
|  |  |  |  | L | 186 |  |  |
|  | | | Retrolenticular part of internal capsule | R | 370 | 641 | .011 |
|  |  |  |  | L | 271 |  |  |
|  | | | Cingulum | R | 221 | 586 | .022 |
|  |  |  |  | L | 365 |  |  |
|  | | | Sagittal stratum | R | 283 | 501 | .017 |
|  |  |  |  | L | 218 |  |  |
|  | | | Cerebral peduncle | R | 175 | 360 | .026 |
|  |  |  |  | L | 185 |  |  |
|  | | | Fornix | R | 149 | 292 | .021 |
|  |  |  |  | L | 143 |  |  |
|  | | | Superior fronto-occipital fasciculus | R | 101 | 196 | .003 |
|  |  |  |  | L | 95 |  |  |
|  | | | Uncinate fasciculus | R | 73 | 131 | .008 |
|  |  |  |  | L | 58 |  |  |

*Note*. Cluster-specific TBSS results for the group comparison neuro-WD vs. healthy controls in NODDI-derived ECVF. For more detailed explanation, see general description above.

**Table S4** **WM regions with altered FA in neuro-WD patients compared with healthy controls**

|  | FA decrease | | | | | | | | | | | | | | |
| --- | --- | --- | --- | --- | --- | --- | --- | --- | --- | --- | --- | --- | --- | --- | --- |
|  | Peak MNI coordinates | | | | | | **WM regions** | | | **Side** | **Voxels** | | **Total voxels** | | **Mean *p*** |
|  | X | | **Y** | **Z** | | |  |  |  |  |  |  |  |  |  |
|  | 32 | | 6 | 4 | | |  | | |  |  | | 4,329 | | *p_min_* = .016 |
|  |  | | | | | | External capsule | | | R | 750 | | 750 | | .027 |
|  |  | | | | | | Anterior corona radiata | | | R | 511 | | 511 | | .037 |
|  |  | | | | | | Retrolenticular part of internal capsule | | | R | 258 | | 258 | | .033 |
|  |  | | | | | | Superior longitudinal fasciculus | | | R | 130 | | 130 | | .036 |
|  |  | | | | | | Sagittal stratum | | | R | 126 | | 126 | | .035 |
|  | -33 | | -8 | | 6 | |  | | |  |  | | 528 | | *p_min_* = .020 |
|  |  | | | | | | External capsule | | | L | 413 | | 413 | | .033 |
| FA increase | | | | | | | | | | | | | | | |
|  | Peak MNI coordinates | | | | | **WM regions** | | **Side** | **Voxels** | | | **Total voxels** | | **Mean *p*** | |
|  | X | | **Y** | **Z** | |  |  |  |  |  |  |  |  |  |  |
|  | -24 | -21 | | 22 | | |  | | |  |  | | 532 | | *p_min_* = .022 |
|  |  | | | | | | Superior corona radiata | | | L | 258 | | 258 | | .032 |
|  |  | | | | | | Posterior limb of internal capsule | | | L | 174 | | 174 | | .036 |
|  | 22 | -8 | | 18 | | |  | | |  |  | | 245 | | *p_min_* = .030 |
|  |  | | | | | | Superior corona radiata | | | R | 160 | | 160 | | .036 |

*Note*. Cluster-specific TBSS results for the group comparison neuro-WD vs. healthy controls in DTI-derived FA. For more detailed explanation, see general description above.

**Table S5 WM regions with increased MD in neuro-WD patients compared with healthy controls**

| Peak MNI coordinates | | | WM regions | Side | Voxels | Total voxels | Mean *p* |
| --- | --- | --- | --- | --- | --- | --- | --- |
| X | **Y** | **Z** |  |  |  |  |  |
| 8 | 21 | -18 |  |  |  | 85,009 | *p_min_* < .001 |
|  | | | Corpus callosum | B | 3,107 | 5,986 | .008 |
|  |  |  |  | G | 1,258 |  |  |
|  |  |  |  | S | 1,621 |  |  |
|  | | | Superior longitudinal fasciculus | R | 1,511 | 2,897 | .002 |
|  |  |  |  | L | 1,386 |  |  |
|  | | | Superior corona radiata | R | 1,473 | 2,855 | .001 |
|  |  |  |  | L | 1,382 |  |  |
|  | | | Anterior corona radiata | R | 1,368 | 2,780 | .004 |
|  |  |  |  | L | 1,412 |  |  |
|  | | | External capsule | R | 1,027 | 2,105 | .004 |
|  |  |  |  | L | 1,078 |  |  |
|  | | | Posterior thalamic radiation | R | 776 | 1,448 | .015 |
|  |  |  |  | L | 672 |  |  |
|  | | | Posterior corona radiata | R | 652 | 1,244 | .005 |
|  |  |  |  | L | 592 |  |  |
|  | | | Posterior limb of internal capsule | R | 496 | 1,015 | .009 |
|  |  |  |  | L | 519 |  |  |
|  | | | Retrolenticular part of internal capsule | R | 537 | 901 | .011 |
|  |  |  |  | L | 364 |  |  |
|  | | | Anterior limb of internal capsule | R | 423 | 814 | .006 |
|  |  |  |  | L | 391 |  |  |
|  | | | Sagittal stratum | R | 396 | 771 | .013 |
|  |  |  |  | L | 375 |  |  |
|  | | | Cingulum | R | 324 | 731 | .005 |
|  |  |  |  | L | 407 |  |  |
|  | | | Cerebral peduncle | R | 130 | 289 | .013 |
|  |  |  |  | L | 159 |  |  |
|  | | | Fornix | R | 171 | 351 | .013 |
|  |  |  |  | L | 180 |  |  |
|  | | | Superior fronto-occipital fasciculus | R | 109 | 206 | < .001 |
|  |  |  |  | L | 97 |  |  |
|  | | | Uncinate fasciculus | R | 74 | 137 | .005 |
|  |  |  |  | L | 63 |  |  |

*Note*. Cluster-specific TBSS results for the group comparison neuro-WD vs. healthy controls in DTI-derived MD. For more detailed explanation, see general description above.

**Fig. S1 Differences in AD and RD between neuro-WD patients and healthy controls.** WM regions with significant clusters of increased (blue) diffusion metrics in neuro-WD patients compared with healthy controls are shown on the study-specific mean WM skeleton (green) on the FMRIB58 FA template (TFCE, FWE-corrected *p* ≤ .05). Results are presented on representative axial slices, ranging from Z = -20 to 30 (in MNI coordinates). The TBSS fill script was used to aid visualization. Images are displayed in accordance with radiological convention, meaning that the left side of the images corresponds to the right hemisphere of the brain and vice versa

AD = axial diffusivity; RD = radial diffusivity; neuro-WD = Wilson disease patients with a neurological phenotype; WM = white matter; FA = fractional anisotropy; TFCE = Threshold-Free Cluster Enhancement; FWE = family-wise error; MNI = Montreal Neurological Institute; TBSS = Tract-Based Spatial Statistics.

**Table S6 WM regions with increased AD in neuro-WD patients compared with healthy controls**

| Peak MNI coordinates | | | WM regions | Side | Voxels | Total voxels | Mean *p* |
| --- | --- | --- | --- | --- | --- | --- | --- |
| X | **Y** | **Z** |  |  |  |  |  |
| -12 | -15 | -15 |  |  |  | 54,536 | *p_min_* < .001 |
|  | | | Corpus callosum | B | 2,873 | 6,313 | .004 |
|  |  |  |  | G | 1,185 |  |  |
|  |  |  |  | S | 2,255 |  |  |
|  | | | Superior corona radiata | R | 1,276 | 2,518 | .002 |
|  |  |  |  | L | 1,242 |  |  |
|  | | | Superior longitudinal fasciculus | R | 1,064 | 2,046 | .005 |
|  |  |  |  | L | 982 |  |  |
|  | | | Anterior corona radiata | R | 892 | 1,621 | .004 |
|  |  |  |  | L | 729 |  |  |
|  | | | External capsule | R | 498 | 1,136 | .007 |
|  |  |  |  | L | 638 |  |  |
|  | | | Posterior corona radiata | R | 588 | 1,083 | .004 |
|  |  |  |  | L | 495 |  |  |
|  | | | Anterior limb of internal capsule | R | 500 | 968 | .002 |
|  |  |  |  | L | 468 |  |  |
|  | | | Posterior limb of internal capsule | R | 706 | 706 | .002 |
|  | | | Cerebral peduncle | R | 307 | 599 | .005 |
|  |  |  |  | L | 292 |  |  |
|  | | | Retrolenticular part of internal capsule | R | 285 | 598 | .005 |
|  |  |  |  | L | 313 |  |  |
|  | | | Posterior thalamic radiation | R | 307 | 558 | .009 |
|  |  |  |  | L | 251 |  |  |
|  | | | Corticospinal tract | R | 161 | 413 | .009 |
|  |  |  |  | L | 252 |  |  |
|  | | | Cingulum | R | 190 | 404 | .008 |
|  |  |  |  | L | 214 |  |  |
|  | | | Superior cerebellar peduncle | R | 179 | 346 | .006 |
|  |  |  |  | L | 167 |  |  |
|  | | | Medial lemniscus | R | 133 | 260 | .007 |
|  |  |  |  | L | 127 |  |  |
|  | | | Superior fronto-occipital fasciculus | R | 108 | 205 | < .001 |
|  |  |  |  | L | 97 |  |  |
|  | | | Fornix | R | 123 | 189 | .009 |
|  |  |  |  | L | 66 |  |  |
|  | | | Cerebellar peduncle | M | 150 | 150 | .013 |
|  | | | Uncinate fasciculus | R | 78 | 141 | .007 |
|  |  |  |  | L | 63 |  |  |
|  | | | Pontine crossing tract |  | 125 | 125 | .010 |
|  | | | Sagittal stratum | R | 58 | 123 | .021 |
|  |  |  |  | L | 65 |  |  |

*Note*. Cluster-specific TBSS results for the group comparison neuro-WD vs. healthy controls in DTI-derived AD. For more detailed explanation, see general description above.

**Table S7 WM regions with increased RD in neuro-WD patients compared with healthy controls**

| Peak MNI coordinates | | | WM regions | Side | Voxels | Total voxels | Mean *p* |
| --- | --- | --- | --- | --- | --- | --- | --- |
| X | **Y** | **Z** |  |  |  |  |  |
| 34 | -10 | 7 |  |  |  | 57,928 | *p_min_* = .001 |
|  |  |  | Anterior corona radiata | R | 1,167 | 2,391 | .009 |
|  |  |  |  | L | 1,224 |  |  |
|  | | | Corpus callosum | B | 1,415 | 2,162 | .017 |
|  |  |  |  | G | 673 |  |  |
|  |  |  |  | S | 74 |  |  |
|  | | | External capsule | R | 1,076 | 2,134 | .008 |
|  |  |  |  | L | 1,058 |  |  |
|  | | | Superior longitudinal fasciculus | R | 874 | 1,771 | .012 |
|  |  |  |  | L | 879 |  |  |
|  | | | Superior corona radiata | R | 700 | 1,467 | .011 |
|  |  |  |  | L | 767 |  |  |
|  | | | Posterior thalamic radiation | R | 622 | 1,120 | .015 |
|  |  |  |  | L | 498 |  |  |
|  | | | Sagittal stratum | R | 499 | 848 | .011 |
|  |  |  |  | L | 349 |  |  |
|  | | | Retrolenticular part of internal capsule | R | 401 | 727 | .010 |
|  |  |  |  | L | 326 |  |  |
|  | | | Posterior corona radiata | R | 350 | 629 | .020 |
|  |  |  |  | L | 279 |  |  |
|  | | | Cingulum | R | 57 | 330 | .020 |
|  |  |  |  | L | 273 |  |  |
|  | | | Fornix | R | 8 | 224 | .013 |
|  |  |  |  | L | 217 |  |  |

*Note*. Cluster-specific TBSS results for the group comparison neuro-WD vs. healthy controls in DTI-derived RD. For more detailed explanation, see general description above.

1.2 **Hep-WD patients vs. healthy controls**

**Table S8 WM regions with increased ISOVF in hep-WD patients compared with healthy controls**

| Peak MNI coordinates | | | WM regions | Side | Voxels | Total voxels | Mean *p* |
| --- | --- | --- | --- | --- | --- | --- | --- |
| X | **Y** | **Z** |  |  |  |  |  |
| -19 | 47 | 1 |  |  |  | 10,793 | *p_min_* = .011 |
|  | | | Corpus callosum | B | 325 | 769 | .030 |
|  |  |  |  | G | 55 |  |  |
|  |  |  |  | S | 389 |  |  |
|  | | | Anterior corona radiata | L | 611 | 611 | .021 |
|  | | | Superior longitudinal fasciculus | L | 589 | 589 | .022 |
|  | | | Superior corona radiata | L | 421 | 421 | .024 |
|  | | | Posterior limb of internal capsule | L | 300 | 300 | .041 |
|  | | | External capsule | L | 254 | 254 | .030 |
|  | | | Anterior limb of internal capsule | L | 205 | 205 | .026 |
|  | | | Retrolenticular part of internal capsule | L | 196 | 196 | .042 |
|  | | | Fornix | L | 128 | 128 | .042 |
|  | | | Posterior corona radiata | L | 122 | 122 | .024 |
|  | | | Sagittal stratum | L | 107 | 107 | .041 |
| 21 | 49 | -7 |  |  |  | 7,283 | *p_min_* = .028 |
|  | | | Corpus callosum | B | 534 | 950 | .038 |
|  |  |  |  | G | 284 |  |  |
|  |  |  |  | S | 132 |  |  |
|  | | | Anterior corona radiata | R | 642 | 642 | .038 |
|  | | | Superior corona radiata | R | 519 | 519 | .040 |
|  | | | Superior longitudinal fasciculus | R | 397 | 397 | .039 |

*Note*. Cluster-specific TBSS results for the group comparison hep-WD vs. healthy controls in NODDI-derived ISOVF. For more detailed explanation, see general description above.

1.3 **WD subgroups: Neuro-WD patients vs. hep-WD patients**

**Table S9 WM regions with decreased NDI in neuro-WD patients compared with hep-WD patients**

| Peak MNI coordinates | | | WM regions | Side | Voxels | Total voxels | Mean *p* |
| --- | --- | --- | --- | --- | --- | --- | --- |
| X | **Y** | **Z** |  |  |  |  |  |
| -31 | -11 | 12 |  |  |  | 2,632 | *p_min_* = .037 |
|  | | | Superior corona radiata | L | 872 | 872 | .040 |
|  | | | External capsule | L | 186 | 186 | .041 |

*Note*. Cluster-specific TBSS results for the group comparison neuro-WD vs. hep-WD in NODDI-derived NDI. For more detailed explanation, see general description above.

**Table S10 WM regions with decreased ODI in neuro-WD patients compared with hep-WD patients**

| Peak MNI coordinates | | | | | WM regions | Side | Voxels | Total voxels | Mean *p* |
| --- | --- | --- | --- | --- | --- | --- | --- | --- | --- |
| X | | **Y** | | **Z** |  |  |  |  |  |
| -25 | | -10 | | 17 |  |  |  | 5,639 | *p_min_* < .001 |
|  | | | | | Corpus callosum | B | 1,315 | 2,076 | .019 |
|  |  |  |  |  |  | G | 474 |  |  |
|  |  |  |  |  |  | S | 287 |  |  |
|  | | | | | Superior corona radiata | L | 802 | 802 | .006 |
|  | | | | | Anterior corona radiata | R | 1 | 502 | .010 |
|  |  |  |  |  |  | L | 501 |  |  |
|  | | | | | Posterior limb of internal capsule | L | 434 | 434 | .009 |
|  | | | | | Posterior corona radiata | L | 323 | 323 | .013 |
|  | | | | | Anterior limb of internal capsule | L | 316 | 316 | .012 |
|  | | | | | Retrolenticular part of internal capsule | L | 292 | 292 | .018 |
| 25 | -10 | | 16 | |  |  |  | 2,079 | *p_min_* = .001 |
|  | | | | | Superior corona radiata | R | 563 | 563 | .011 |
|  | | | | | Anterior corona radiata | R | 457 | 457 | .012 |
|  | | | | | Posterior corona radiata | R | 292 | 292 | .018 |
|  | | | | | Posterior limb of internal capsule | R | 244 | 244 | .011 |
|  | | | | | Retrolenticular part of internal capsule | R | 163 | 163 | .016 |
|  | | | | | Anterior limb of internal capsule | R | 103 | 103 | .017 |

*Note*. Cluster-specific TBSS results for the group comparison neuro-WD vs. hep-WD in NODDI-derived ODI. For more detailed explanation, see general description above.

**Table S11 WM regions with increased ISOVF in hep-WD patients compared with neuro-WD patients**

| Peak MNI coordinates | | | WM regions | Side | Voxels | Total voxels | Mean *p* |
| --- | --- | --- | --- | --- | --- | --- | --- |
| X | **Y** | **Z** |  |  |  |  |  |
| -13 | 25 | 48 |  |  |  | 13,856 | *p_min_* = .016 |
|  | | | Corpus callosum | B | 726 | 1,449 | .030 |
|  |  |  |  | G | 723 |  |  |
|  | | | Anterior corona radiata | R | 421 | 1,042 | .041 |
|  |  |  |  | L | 621 |  |  |
|  | | | Anterior limb of internal capsule | R | 321 | 667 | .037 |
|  |  |  |  | L | 346 |  |  |
|  | | | External capsule | R | 221 | 534 | .040 |
|  |  |  |  | L | 313 |  |  |
|  | | | Superior corona radiata | R | 284 | 521 | .041 |
|  |  |  |  | L | 237 |  |  |
|  | | | Posterior limb of internal capsule | R | 270 | 482 | .040 |
|  |  |  |  | L | 212 |  |  |
|  | | | Cerebellar peduncle | M | 337 | 337 | .038 |
|  | | | Cerebral peduncle | R | 329 | 329 | .033 |
|  | | | Posterior corona radiata | L | 125 | 125 | .039 |

*Note*. Cluster-specific TBSS results for the group comparison neuro-WD vs. hep-WD in NODDI-derived ISOVF. For more detailed explanation, see general description above.

**Table S12 WM regions with increased ECVF in neuro-WD patients compared with hep-WD patients**

| Peak MNI coordinates | | | | | WM regions | Side | Voxels | Total voxels | Mean *p* |
| --- | --- | --- | --- | --- | --- | --- | --- | --- | --- |
| X | | **Y** | | **Z** |  |  |  |  |  |
| -29 | | -2 | | 16 |  |  |  | 6,444 | *p_min_* = .025 |
|  | | | | | Superior corona radiata | L | 1,127 | 1,127 | .032 |
|  | | | | | Anterior corona radiata | L | 425 | 425 | .037 |
|  | | | | | External capsule | L | 342 | 342 | .034 |
|  | | | | | Superior longitudinal fasciculus | L | 270 | 270 | .041 |
|  | | | | | Posterior corona radiata | L | 190 | 190 | .039 |
|  | | | | | Anterior limb of internal capsule | L | 159 | 159 | .034 |
|  | | | | | Posterior limb of internal capsule | L | 102 | 102 | .039 |
| 24 | -15 | | 34 | |  |  |  | 2,273 | *p_min_* = .041 |
|  | | | | | Superior corona radiata | R | 784 | 784 | .044 |
|  | | | | | Anterior corona radiata | R | 175 | 175 | .046 |
|  | | | | | Posterior corona radiata | R | 152 | 152 | .045 |
|  | | | | | External capsule | R | 138 | 138 | .045 |

*Note*. Cluster-specific TBSS results for the group comparison neuro-WD vs. hep-WD in NODDI-derived ECVF. For more detailed explanation, see general description above.

**Table S13 WM regions with increased MD in neuro-WD patients compared with hep-WD patients**

| Peak MNI coordinates | | | | | WM regions | Side | Voxels | Total voxels | Mean *p* |
| --- | --- | --- | --- | --- | --- | --- | --- | --- | --- |
| X | | **Y** | | **Z** |  |  |  |  |  |
| -28 | | -2 | | 19 |  |  |  | 6,928 | *p_min_* = .025 |
|  | | | | | Superior corona radiata | L | 1,287 | 1,287 | .031 |
|  | | | | | Corpus callosum | B | 851 | 851 | .041 |
|  | | | | | Anterior corona radiata | L | 480 | 480 | .040 |
|  | | | | | External capsule | L | 408 | 408 | .034 |
|  | | | | | Superior longitudinal fasciculus | L | 343 | 343 | .040 |
|  | | | | | Posterior corona radiata | L | 223 | 223 | .036 |
|  | | | | | Anterior limb of internal capsule | L | 140 | 140 | .033 |
|  | | | | | Posterior limb of internal capsule | L | 113 | 113 | .038 |
| 24 | -18 | | 34 | |  |  |  | 6,381 | *p_min_* = .032 |
|  | | | | | Superior corona radiata | R | 1,296 | 1,296 | .036 |
|  | | | | | Superior longitudinal fasciculus | R | 543 | 543 | .040 |
|  | | | | | Anterior corona radiata | R | 347 | 347 | .041 |
|  | | | | | Posterior corona radiata | R | 260 | 260 | .039 |
|  | | | | | External capsule | R | 219 | 219 | .038 |
|  | | | | | Posterior limb of internal capsule | R | 124 | 124 | .040 |
|  | | | | | Anterior limb of internal capsule | R | 116 | 116 | .039 |

*Note*. Cluster-specific TBSS results for the group comparison neuro-WD vs. hep-WD in DTI-derived MD. For more detailed explanation, see general description above.

**Fig. S2 Differences in AD and RD between neuro-WD and hep-WD patients.** WM regions with significant clusters of increased (blue) diffusion metrics in neuro-WD patients compared with hep-WD patients are shown on the study-specific mean WM skeleton (green) on the FMRIB58 FA template (TFCE, FWE-corrected *p* ≤ .05). Results are presented on representative axial slices, ranging from Z = -20 to 30 (in MNI coordinates). The TBSS fill script was used to aid visualization. Images are displayed in accordance with radiological convention, meaning that the left side of the images corresponds to the right hemisphere of the brain and vice versa

AD = axial diffusivity; RD = radial diffusivity; neuro-WD = Wilson disease patients with a neurological phenotype; hep-WD = Wilson disease patients with a hepatic phenotype; WM = white matter; FA = fractional anisotropy; TFCE = Threshold-Free Cluster Enhancement; FWE = family-wise error; MNI = Montreal Neurological Institute; TBSS = Tract-Based Spatial Statistics

**Table S14 WM regions with increased AD in neuro-WD patients compared with hep-WD patients**

| Peak MNI coordinates | | | WM regions | Side | Voxels | Total voxels | Mean *p* |
| --- | --- | --- | --- | --- | --- | --- | --- |
| X | **Y** | **Z** |  |  |  |  |  |
| -24 | -9 | 15 |  |  |  | 17,031 | *p_min_* < .001 |
|  | | | Corpus callosum | B | 1,971 | 4,028 | .017 |
|  |  |  |  | G | 821 |  |  |
|  |  |  |  | S | 1,236 |  |  |
|  | | | Superior corona radiata | R | 1,146 | 2,350 | .009 |
|  |  |  |  | L | 1,204 |  |  |
|  | | | Anterior corona radiata | R | 714 | 1,445 | .010 |
|  |  |  |  | L | 731 |  |  |
|  | | | Posterior limb of internal capsule | R | 435 | 1,060 | .011 |
|  |  |  |  | L | 625 |  |  |
|  | | | Posterior corona radiata | R | 424 | 805 | .009 |
|  |  |  |  | L | 381 |  |  |
|  | | | Anterior limb of internal capsule | R | 399 | 802 | .010 |
|  |  |  |  | L | 403 |  |  |
|  | | | Superior longitudinal fasciculus | R | 615 | 677 | .023 |
|  |  |  |  | L | 62 |  |  |
|  | | | Retrolenticular part of internal capsule | R | 252 | 637 | .013 |
|  |  |  |  | L | 385 |  |  |
|  | | | External capsule | R | 122 | 357 | .014 |
|  |  |  |  | L | 235 |  |  |
|  | | | Cerebral peduncle | L | 235 | 235 | .035 |
|  | | | Superior fronto-occipital fasciculus | R | 106 | 203 | .005 |
|  |  |  |  | L | 97 |  |  |
|  | | | Posterior thalamic radiation | R | 116 | 202 | .019 |
|  |  |  |  | L | 86 |  |  |
|  | | | Sagittal stratum | L | 112 | 112 | .026 |

*Note*. Cluster-specific TBSS results for the group comparison neuro-WD vs. hep-WD in DTI-derived AD. For more detailed explanation, see general description above.

**2. Cluster-specific statistics: Correlation analyses**

2.1 **Correlations between NDI and clinical scores**

**Table S15 WM regions where NDI decreased with higher UWDRS-N scores**

| Peak MNI coordinates | | | WM regions | Side | Voxels | Total voxels | Mean *p* |
| --- | --- | --- | --- | --- | --- | --- | --- |
| X | **Y** | **Z** |  |  |  |  |  |
| 38 | -8 | 32 |  |  |  | 19,128 | *p_min_* = .035 |
|  | | | Corpus callosum | B | 1,478 | 2,257 | .041 |
|  |  |  |  | G | 363 |  |  |
|  |  |  |  | S | 416 |  |  |
|  | | | Superior corona radiata | R | 863 | 1,893 | .040 |
|  |  |  |  | L | 1,030 |  |  |
|  | | | Superior longitudinal fasciculus | R | 554 | 1,085 | .042 |
|  |  |  |  | L | 531 |  |  |
|  | | | Anterior corona radiata | R | 275 | 791 | .041 |
|  |  |  |  | L | 516 |  |  |
|  | | | External capsule | R | 516 | 752 | .041 |
|  |  |  |  | L | 236 |  |  |
|  | | | Posterior corona radiata | R | 475 | 727 | .040 |
|  |  |  |  | L | 252 |  |  |
|  | | | Posterior limb of internal capsule | R | 338 | 616 | .041 |
|  |  |  |  | L | 278 |  |  |
|  | | | Posterior thalamic radiation | R | 176 | 566 | .042 |
|  |  |  |  | L | 390 |  |  |
|  | | | Anterior limb of internal capsule | R | 321 | 514 | .041 |
|  |  |  |  | L | 193 |  |  |
|  | | | Retrolenticular part of internal capsule | R | 256 | 363 | .042 |
|  |  |  |  | L | 107 |  |  |
|  | | | Corticospinal tract | R | 107 | 343 | .044 |
|  |  |  |  | L | 236 |  |  |
|  | | | Medial lemniscus | R | 137 | 301 | .040 |
|  |  |  |  | L | 164 |  |  |
|  | | | Cerebral peduncle | R | 171 | 253 | .044 |
|  |  |  |  | L | 82 |  |  |
|  | | | Pontine crossing tract |  | 206 | 206 | .044 |
|  | | | Superior cerebellar peduncle | R | 91 | 180 | .041 |
|  |  |  |  | L | 89 |  |  |
|  | | | Cerebellar peduncle | M | 125 | 125 | .045 |
|  | | | Superior fronto-occipital fasciculus | R | 48 | 118 | .041 |
|  |  |  |  | L | 70 |  |  |
| -30 | -56 | -1 |  |  |  | 427 | *p_min_* = .049 |
|  | | | Posterior thalamic radiation | L | 113 | 113 | .049 |

*Note*. Cluster-specific TBSS results for the voxel-wise correlations between NODDI-derived NDI and UWDRS-N scores in the WD cohort. For more detailed explanation, see general description above.

**Table S16** **WM regions where NDI decreased with poorer TMTA performance (controlled for UWDRS-N scores)**

| Peak MNI coordinates | | | WM regions | Side | Voxels | Total voxels | Mean *p* |
| --- | --- | --- | --- | --- | --- | --- | --- |
| X | **Y** | **Z** |  |  |  |  |  |
| -4 | 19 | 17 |  |  |  | 45,922 | *p_min_* = .009 |
|  | | | Corpus callosum | B | 2,066 | 3,696 | .018 |
|  |  |  |  | G | 1,257 |  |  |
|  |  |  |  | S | 373 |  |  |
|  | | | Anterior corona radiata | R | 1,182 | 2,166 | .020 |
|  |  |  |  | L | 984 |  |  |
|  | | | Superior longitudinal fasciculus | R | 753 | 1,547 | .021 |
|  |  |  |  | L | 794 |  |  |
|  | | | External capsule | R | 576 | 1,210 | .019 |
|  |  |  |  | L | 634 |  |  |
|  | | | Superior corona radiata | R | 630 | 937 | .027 |
|  |  |  |  | L | 307 |  |  |
|  | | | Posterior corona radiata | R | 370 | 634 | .024 |
|  |  |  |  | L | 264 |  |  |
|  | | | Anterior limb of internal capsule | R | 375 | 624 | .020 |
|  |  |  |  | L | 249 |  |  |
|  | | | Retrolenticular part of internal capsule | R | 246 | 480 | .023 |
|  |  |  |  | L | 234 |  |  |
|  | | | Sagittal stratum | R | 236 | 414 | .024 |
|  |  |  |  | L | 178 |  |  |
|  | | | Posterior thalamic radiation | R | 169 | 334 | .034 |
|  |  |  |  | L | 165 |  |  |
|  | | | Fornix | R | 95 | 152 | .030 |
|  |  |  |  | L | 57 |  |  |
|  | | | Uncinate fasciculus | R | 78 | 117 | .019 |
|  |  |  |  | L | 39 |  |  |

*Note*. Cluster-specific TBSS results for the voxel-wise correlations between NODDI-derived NDI and TMTA scores in the WD cohort. For more detailed explanation, see general description above.

**Fig. S3 Significant correlations between NDI and neuropsychological scores.** WM regions with significant clusters of decreased NDI with decreasing neuropsychological performance on the SDMT and TMTB (blue; not controlled for UWDRS-N scores) in WD patients are shown on the study-specific mean WM skeleton (green) on the FMRIB58 FA template (TFCE, FWE-corrected *p* ≤ .05). Results are presented on three representative slices (MNI coordinates X, Y, and Z). The TBSS fill script was used to aid visualization. The images are displayed in accordance with radiological convention, meaning that the left side of the images corresponds to the right hemisphere of the brain and vice versa

NDI = neurite density index; SDMT = Symbol Digit Modalities Test; TMTB = Trail Making Test Part B; WM = white matter; UWDRS-N = Unified Wilson’s Disease Rating Scale neurological subscale; WD = Wilson disease; FA = fractional anisotropy; TFCE = Threshold-Free Cluster Enhancement; FWE = family-wise error; MNI = Montreal Neurological Institute; TBSS = Tract-Based Spatial Statistics.

**Table S17 WM regions where NDI decreased with poorer SDMT performance**

| Peak MNI coordinates | | | WM regions | Side | Voxels | Total voxels | Mean *p* |
| --- | --- | --- | --- | --- | --- | --- | --- |
| X | **Y** | **Z** |  |  |  |  |  |
| 42 | -5 | -29 |  |  |  | 39,596 | *p_min_* = .025 |
|  | | | Corpus callosum | B | 1,623 | 3,782 | .035 |
|  |  |  |  | G | 1,194 |  |  |
|  |  |  |  | S | 965 |  |  |
|  | | | Superior corona radiata | R | 914 | 1,732 | .038 |
|  |  |  |  | L | 818 |  |  |
|  | | | External capsule | R | 920 | 1,591 | .039 |
|  |  |  |  | L | 671 |  |  |
|  | | | Anterior corona radiata | R | 981 | 1,451 | .037 |
|  |  |  |  | L | 470 |  |  |
|  | | | Superior longitudinal fasciculus | R | 585 | 1,107 | .037 |
|  |  |  |  | L | 522 |  |  |
|  | | | Posterior thalamic radiation | R | 266 | 830 | .037 |
|  |  |  |  | L | 564 |  |  |
|  | | | Posterior corona radiata | R | 455 | 787 | .036 |
|  |  |  |  | L | 332 |  |  |
|  | | | Anterior limb of internal capsule | R | 389 | 651 | .040 |
|  |  |  |  | L | 262 |  |  |
|  | | | Sagittal stratum | R | 430 | 649 | .035 |
|  |  |  |  | L | 219 |  |  |
|  | | | Retrolenticular part of internal capsule | R | 323 | 456 | .037 |
|  |  |  |  | L | 133 |  |  |
|  | | | Posterior limb of internal capsule | R | 191 | 361 | .042 |
|  |  |  |  | L | 170 |  |  |
|  | | | Superior cerebellar peduncle | R | 116 | 198 | .045 |
|  |  |  |  | L | 82 |  |  |
|  | | | Fornix | R | 174 | 187 | .039 |
|  |  |  |  | L | 13 |  |  |
|  | | | Cerebral peduncle | R | 157 | 175 | .048 |
|  |  |  |  | L | 18 |  |  |
|  | | | Pontine crossing tract |  |  | 154 | .045 |
|  | | | Medial lemniscus | R | 68 | 128 | .046 |
|  |  |  |  | L | 60 |  |  |

*Note*. Cluster-specific TBSS results for the voxel-wise correlations between NODDI-derived NDI and SDMT scores in the WD cohort. For more detailed explanation, see general description above.

**Table S18** **WM regions where NDI decreased with poorer TMTB performance**

| Peak MNI coordinates | | | WM regions | Side | Voxels | Total voxels | Mean *p* |
| --- | --- | --- | --- | --- | --- | --- | --- |
| X | **Y** | **Z** |  |  |  |  |  |
| -35 | -26 | 34 |  |  |  | 27,456 | *p_min_* = .010 |
|  | | | Corpus callosum | B | 1,769 | 2,540 | .022 |
|  |  |  |  | G | 461 |  |  |
|  |  |  |  | S | 310 |  |  |
|  | | | Superior corona radiata | R | 1,052 | 2,115 | .019 |
|  |  |  |  | L | 1,063 |  |  |
|  | | | Anterior corona radiata | R | 908 | 1,397 | .022 |
|  |  |  |  | L | 489 |  |  |
|  | | | External capsule | R | 717 | 1,303 | .034 |
|  |  |  |  | L | 586 |  |  |
|  | | | Superior longitudinal fasciculus | R | 508 | 1,114 | .024 |
|  |  |  |  | L | 606 |  |  |
|  | | | Posterior thalamic radiation | R | 361 | 883 | .025 |
|  |  |  |  | L | 522 |  |  |
|  | | | Posterior corona radiata | R | 493 | 758 | .021 |
|  |  |  |  | L | 265 |  |  |
|  | | | Anterior limb of internal capsule | R | 343 | 640 | .023 |
|  |  |  |  | L | 297 |  |  |
|  | | | Posterior limb of internal capsule | R | 298 | 534 | .024 |
|  |  |  |  | L | 236 |  |  |
|  | | | Retrolenticular part of internal capsule | R | 282 | 410 | .029 |
|  |  |  |  | L | 128 |  |  |
|  | | | Superior fronto-occipital fasciculus | R | 94 | 154 | .019 |
|  |  |  |  | L | 60 |  |  |
|  | | | Sagittal stratum | R | 86 | 136 | .041 |
|  |  |  |  | L | 50 |  |  |

*Note*. Cluster-specific TBSS results for the voxel-wise correlations between NODDI-derived NDI and TMTB scores in the WD cohort. For more detailed explanation, see general description above.

2.2 **Correlations between FA and clinical scores**

**Table S19 WM regions where FA decreased with higher UWDRS-N scores**

| Peak MNI coordinates | | | WM regions | Side | Voxels | Total voxels | Mean *p* |
| --- | --- | --- | --- | --- | --- | --- | --- |
| X | **Y** | **Z** |  |  |  |  |  |
| -17 | 22 | 25 |  |  |  | 13,341 | *p_min_* = .028 |
|  | | | Corpus callosum | B | 1,304 | 1,997 | .034 |
|  |  |  |  | G | 641 |  |  |
|  |  |  |  | S | 52 |  |  |
|  | | | Anterior corona radiata | R | 708 | 1,439 | .035 |
|  |  |  |  | L | 731 |  |  |
|  | | | External capsule | L | 746 | 746 | .043 |
|  | | | Superior corona radiata | R | 370 | 559 | .034 |
|  |  |  |  | L | 189 |  |  |
|  | | | Superior longitudinal fasciculus | L | 525 | 525 | .034 |
|  | | | Medial lemniscus | R | 114 | 248 | .040 |
|  |  |  |  | L | 134 |  |  |
|  | | | Superior cerebellar peduncle | R | 146 | 237 | .040 |
|  |  |  |  | L | 91 |  |  |
| -26 | -74 | 1 |  |  |  | 204 | *p_min_* = .046 |
| -30 | -48 | 16 |  |  |  | 125 | *p_min_* = .047 |
|  | | | Posterior thalamic radiation | L | 103 | 103 | .048 |

*Note*. Cluster-specific TBSS results for the voxel-wise correlations between DTI-derived FA and UWDRS-N scores in the WD cohort. For more detailed explanation, see general description above.

**Table S20 WM regions where FA decreased with poorer TMTA performance (controlled for UWDRS-N scores)**

| Peak MNI coordinates | | | WM regions | Side | Voxels | Total voxels | Mean *p* |
| --- | --- | --- | --- | --- | --- | --- | --- |
| X | **Y** | **Z** |  |  |  |  |  |
| 5 | -34 | -26 |  |  |  | 16,781 | *p_min_* = .019 |
|  |  |  | Corpus callosum | B | 1,383 | 2,341 | .035 |
|  |  |  |  | G | 557 |  |  |
|  |  |  |  | S | 401 |  |  |
|  |  |  | Anterior corona radiata | R | 814 | 1,804 | .034 |
|  |  |  |  | L | 990 |  |  |
|  |  |  | External capsule | R | 430 | 1,114 | .035 |
|  |  |  |  | L | 684 |  |  |
|  |  |  | Superior corona radiata | R | 260 | 659 | .035 |
|  |  |  |  | L | 399 |  |  |
|  |  |  | Posterior thalamic radiation | L | 599 | 599 | .042 |
|  |  |  | Cerebral peduncle | R | 105 | 449 | .029 |
|  |  |  |  | L | 344 |  |  |
|  |  |  | Superior cerebellar peduncle | R | 185 | 362 | .028 |
|  |  |  |  | L | 177 |  |  |
|  |  |  | Fornix | R | 161 | 349 | .035 |
|  |  |  |  | L | 188 |  |  |
|  |  |  | Retrolenticular part of internal capsule | R | 93 | 305 | .035 |
|  |  |  |  | L | 212 |  |  |
|  |  |  | Posterior limb of internal capsule | R | 86 | 269 | .032 |
|  |  |  |  | L | 183 |  |  |
|  |  |  | Medial lemniscus | R | 98 | 236 | .028 |
|  |  |  |  | L | 138 |  |  |
|  |  |  | Cingulum | L | 214 | 214 | .035 |
|  |  |  | Sagittal stratum | R | 21 | 208 | .035 |
|  |  |  |  | L | 187 |  |  |
|  |  |  | Posterior corona radiata | R | 107 | 188 | .037 |
|  |  |  |  | L | 81 |  |  |
|  |  |  | Corticospinal tract | L | 135 | 135 | .028 |
|  |  |  | Pontine crossing tract |  |  | 125 | .030 |
|  |  |  | Anterior limb of internal capsule | R | 32 | 117 | .039 |
|  |  |  |  | L | 85 |  |  |

*Note*. Cluster-specific TBSS results for the voxel-wise correlations between DTI-derived FA and TMTA scores in the WD cohort. For more detailed explanation, see general description above.

**Fig. S4 Significant correlations between FA and neuropsychological scores.** WM regions with significant clusters of decreased FA with decreasing TMTB performance (blue; not controlled for UWDRS-N scores) in WD patients are shown on the study-specific mean WM skeleton (green) on the FMRIB58 FA template (TFCE, FWE-corrected *p* ≤ .05). Results are presented on three representative slices (MNI coordinates X, Y, and Z). The TBSS fill script was used to aid visualization. The images are displayed in accordance with radiological convention, meaning that the left side of the images corresponds to the right hemisphere of the brain and vice versa

FA = fractional anisotropy; WM = white matter; TMTB = Trail Making Test Part B; UWDRS-N = Unified Wilson’s Disease Rating Scale neurological subscale; WD = Wilson disease; TFCE = Threshold-Free Cluster Enhancement; FWE = family-wise error; MNI = Montreal Neurological Institute; TBSS = Tract-Based Spatial Statistics.

**Table S21** **WM regions where FA decreased with poorer TMTB performance (not controlled for UWDRS-N scores)**

| Peak MNI coordinates | | | WM regions | Side | Voxels | Total voxels | Mean *p* |
| --- | --- | --- | --- | --- | --- | --- | --- |
| X | **Y** | **Z** |  |  |  |  |  |
| 1 | -30 | -14 |  |  |  | 288 | *p_min_* = .039 |
| -11 | -16 | 29 |  |  |  | 208 | *p_min_* = .042 |
|  |  |  | Corpus callosum | B | 204 | 204 | .047 |
| 12 | -17 | 30 |  |  |  | 176 | *p_min_* = .040 |
|  | | | Corpus callosum | B | 163 | 163 | .045 |

*Note*. Cluster-specific TBSS results for the voxel-wise correlations between DTI-derived FA and TMTB scores in the WD cohort. For more detailed explanation, see general description above.
